# Subtotal Cholecystectomy Results in High Peri-operative Morbidity and Its Risk-Profile Should be Emphasised During Consent

**DOI:** 10.1007/s00268-022-06737-0

**Published:** 2022-10-08

**Authors:** James Lucocq, David Hamilton, John Scollay, Pradeep Patil

**Affiliations:** Department of General and Upper GI Surgery, Ninewells Hospital, Dundee, Scotland

## Abstract

**Background:**

Subtotal cholecystectomy aims to reduce the likelihood of bile duct injury but risks a multitude of less severe, yet significant complications. The primary aim of the present study was to report peri-operative outcomes of subtotal laparoscopic cholecystectomy (SLC) relative to total laparoscopic cholecystectomy (TLC) to inform the consent process.

**Method:**

All laparoscopic cholecystectomies between 2015 and 2020 in one health board were included. The peri-operative outcomes of SLC (n = 87) and TLC (n = 2650) were reported. Pre-operative variables were compared between the two groups to identify risk factors for SLC. The outcomes between the SLC and TLC were compared using univariate, multivariate and propensity analysis.

**Results:**

Risk factors for SLC included higher age, male gender, cholecystitis, increased biliary admissions, ERCP, cholecystostomy and emergency cholecystectomy. Following SLC, rates of post-operative complication (45.9%), imaging (37.9%) intervention (28.7%) and readmission (29.9%) were significant. The risk profile was vastly heightened compared to that of TLC: intra-operative complications (RR 9.0; *p* < 0.001), post-operative complications [bile leak (RR 58.9; *p* < 0.001), collection (RR 12.2; *p* < 0.001), retained stones (RR 7.2; *p* < 0.001) and pneumonia (RR 5.4; *p* < 0.001)], post-operative imaging (RR 4.4; *p* < 0.001), post-operative intervention (RR 12.3; *p* < 0.001), prolonged PLOS (RR 11.3; *p* < 0.001) and readmission (RR 4.5; *p* < 0.001). The findings were consistent using multivariate logistic regression and propensity analysis.

**Conclusion:**

The relative morbidity associated with SLC is significant and high-risk patients should be counselled for the peri-operative morbidity of subtotal cholecystectomy.

## Introduction

Laparoscopic cholecystectomy (LC) is the gold standard treatment for symptomatic gallstone disease and is the most frequently performed operation in the UK; with 409 612 cholecystectomies being reported in the UK over a recent 5 year period [1, 2]. Patients undergoing LC represent a heterogeneous group with different intra-operative challenges. In cases of severe inflammation distorting the cholecystohepatic triangle, the critical view of safety cannot be achieved. Proceeding with a complete cholecystectomy in such a scenario may risk severe complications, the most notable being bile duct injury. In such cases, the surgeon may opt to perform salvage techniques such as a subtotal cholecystectomy (SLC).[3–7]. Rates of BDI are low following SLC (0.08%) and have demonstrated a decline coinciding with increasing utilisation of SLC as a salvage technique [8, 9]. A multitude of less severe, yet significant complications (e.g. bile leak, biliary fistula, remnant cholecystitis) follow SLC and are deemed acceptable relative to BDI.

The identification of pre-operative patient risk factors for SLC and the acknowledgement of the likely peri-operative course are particularly important in an individualised patient consent process. The peri-operative morbidity of SLC relative to total laparoscopic cholecystectomy (TLC) has not been investigated, yet has implications for consent in high-risk individuals and should be recognised to inform surgical decision-making.

The aim of the present study was to report the peri-operative outcomes of subtotal laparoscopic cholecystectomy (SLC) relative to total laparoscopic cholecystectomy (TLC) and to identify risk factors for STC.

## Method

### Population cohort

LC performed for biliary pathology across three training surgical units between January 2015 and January 2020 under the care of 25 general surgical consultants were included in the study. The surgical units were located in a defined geographical region with a stable population of more than 490,000 people. Ethical approval was granted by the regional information governance committee.

### Data

Data were collected retrospectively from multiple regional databases using a deterministic records-linkage methodology. Patients were tracked between databases using a unique patient identifier. Pre-operative data included demographics, American society of anaesthesiology score (ASA), number of admissions, indication, pre-operative ERCP and pre-operative cholecystectomy. Operative data included intra-operative complications, use of drains and operative technique (e.g. SLC vs. TLC). All patients were followed up for 100 days for post-operative outcomes. A prolonged post-operative length of stay (PLOS) was defined as an in-patient stay of at least 3 days following the cholecystectomy.

### Analysis

The pre-operative, intra-operative and post-operative outcomes were reported for both SLC and TLC. To compare the peri-operative course of SLC and TLC, outcomes were compared using both univariate and multivariate analysis. Univariate tests included Chi-squared, Fisher exact and Mann–Whitney U tests. Multivariate logistic regression models were created to investigate associations between SLC and adverse operative and post-operative outcomes after adjusting for pre-operative variables. These models adjusted for the following pre-operative variables: sex, age (<40;40–60; >60), number of biliary-related admissions (1;2; ≥3), ASA (1;2 ≥3), cholecystitis, gallstone pancreatitis, choledocholithiasis, pre-operative ERCP and pre-operative cholecystostomy. In the multivariate logistic regression the odds ratios of SLC for each adverse outcome were reported. Both the univariate analysis and multivariate logistic regression were conducted using STATA/IC 2019 statistical package.

Propensity score matching was conducted in R Studio 2022.02.1 using the ‘MatchIt’ package. SLC patients were matched with comparable TLC patients using pre-operative variables using ‘nearest neighbour’ matching. Once matched multivariate linear regressions were used to determine the relationship between SLC and peri-operative outcomes after adjusting for pre-operative covariates. The coefficient of subtotal as an independent variable for each adverse outcome was reported.

## Results

Over the 5 year period from January 2015 to January 2020, 87 SLC and 2650 TLC were performed. SLC represented 3.2% of all laparoscopic cholecystectomies (median age, 62 years; M:F, 1:1.1; median ASA, 2). In all cases of SLC, SLC was not intended pre-operatively and initial dissection to achieve the critical view of safety was attempted until deemed unsafe. The SLC was performed by the consultant surgeon in all cases.

The most common reasons for performing a SLC include adhesions in cholecystohepatic triangle (80.5%), intra-hepatic gallbladder (11.5%), empyema (8.0%) and impacted stone in Hartmann’s pouch (6.9%) (Table 1). In 73 cases (83.9%) the gallbladder stump was left open (fenestrated), in 5 cases (5.7%) the stump was closed (reconstituting) and in the remaining cases the status of the stump was unclear. In 62 cases (71.3%) the posterior wall of the gallbladder was left in situ.

**Table 1:** Reasons for SLC

Reasons for SLC	Number (%)
Adhesions in cholecystohepatic triangle	70 (80.5)
Intra-hepatic gallbladder	10 (11.5)
Empyema	7 (8.0)
Impacted stone in Hartmann’s pouch	6 (6.9)
Duodenal adhesions	5 (5.7)
Cholecystoduodenal adhesions	5 (5.7)
Mirizzi syndrome	4 (4.6)
Gallbladder mass	3 (3.4)
Abnormal ductal anatomy	3 (3.4)
Cholecystoduodenal fistula	3 (3.4)
Cholecystocolonic adhesions	2 (2.3)
Cholecystocolonic fistula	1 (1.1)

Fifty-two patients (59.8%) underwent a SLC during elective surgery and 35 patients (40.2%) during emergency surgery.

Of the 52 patients that underwent an elective SLC, 39 patients (75.0%) had a pre-operative diagnosis of acute cholecystitis. Twenty-eight patients (53.8) had one previous biliary-related admission, 9 (17.3%) had two admissions and 7 (13.5%) with ≥3 admissions. Seventeen patients (32.7%) had a previous ERCP and six patients (11.5%) a previous cholecystostomy.

Of the 35 patients that underwent an emergency SLC, 30 patients (85.7%) had a pre-operative diagnosis of acute cholecystitis, 4 patients (11.4%) had gallstone pancreatitis and one patient (2.9%) had simple biliary colic. Ten patients (28.6%) had more than one emergency admission. Fourteen patients (40.0%) had a previous ERCP and two patients (5.7%) a previous cholecystostomy.

### Risk factors for SLC

Patients who underwent SLC were older (62 vs. 52 years) and more likely to be male (*p* < 0.001) (Table 2). SLC patients were more likely to have a pre-operative ERCP and cholecystostomy (*p* < 0.001) and were more likely to have cholecystitis, wall thickening and pericholecystic fluid on imaging (*p* < 0.001). SLC patients had more admissions prior to cholecystectomy (*p* < 0.001) and were more likely to have a previous abandoned LC (*p* = 0.002). SLC were more likely during an emergency cholecystectomy (*p* < 0.001) (Table 3).

**Table 2:** Pre-operative data, SLC and TLC

	TLC (n = 2650)	SLC (n = 87)	*p* value
Median age (range), years	52 (13–92)	62 (18–83)	<0.001
Male:Female	1:2.9	1:1.1	<0.001
*ASA*			
1	904 (34.1)	25 (28.7)	0.30
2	1496 (56.4)	51 (58.6)	0.69
≥3	251 (9.5)	11 (12.6)	0.32
Indication, (%)			
Biliary colic	1468 (55.4)	9 (10.3)	<0.001
Cholecystitis	884 (33.4)	69 (79.3)	<0.001
Gallstone pancreatitis	173 (6.5)	6 (6.9)	0.89
Other (including polyps, biliary dyskinesia etc.)	125 (4.7)	3 (3.4)	0.58
*Imaging, (%)*			
USS abdomen	2541 (95.9)	80 (92.0)	0.07
MRCP	858 (32.4)	49 (56.3)	<0.001
CT abdomen/pelvis	358 (13.5)	36 (41.4)	<0.001
*Pre-operative radiological findings, (%)*			
Thickened gallbladder wall	856 (32.3)	54 (62.1)	<0.001
Pericholecystic fluid	356 (13.4)	32 (36.8)	<0.001
CBD stones	237 (8.9)	24 (27.6)	<0.001
*Pre-operative intervention*			
ERCP, (%)	235 (8.9)	31 (35.6)	<0.001
Cholecystostomy, (%)	30 (1.1)	8 (9.2)	<0.001
*Number of biliary-related admissions, (%)*			
1	1143 (43.1)	52 (59.8)	0.002
2	212 (8.0)	16 (18.4)	<0.001
≥3	55 (2.1)	11 (12.6)	<0.001
Previous failed cholecystectomy	16 (0.6)	3 (3.4)	0.002

**Table 3:** Operative data of SLC and TLC; univariate comparative analysis

	TLC	SLC	Relative risk in SLC	*p *value
Emergency:elective operation	1:3.7	1:1.5	–	<0.001
Days from admission to operation, median (emergency subgroup)	3	2	–	0.54
Median Operative time, minutes	73	125	–	<0.001
Intra-operative cholangiogram, (%)	32 (1.2)	8 (9.1)	7.6	<0.001
Detection of stone	5 (0.2)	0 (0.0)	–	1
Unplanned bile duct exploration	1 (0.04)	0 (0.0)	–	1
Placement of intra-operative drain, (%)	114 (4.3)	80 (92.0)	21.4	<0.001
Intra-operative complications	34 (1.3)	10 (11.5)	9.0	<0.001
Bile leak	12 (0.5)	*	–	–
Haemorrhage	15 (0.6)	5 (5.7)	9.5	<0.001
Bowel injury	3 (0.1)	4 (5.0)	40.6	<0.001
Bile duct injury	4 (0.2)	0 (0.0)	–	1
Other	9 (0.3)	1 (1.1)	3.4	0.22

^*^intra-operative bile leak in all cases and not regarded as complication

### Peri-operative morbidity of SLC

Operative and post-operative outcomes of SLC are reported in Tables 3 and 4, respectively. Intra-operative complications were more common in the SLC group (RR 9.0; *p* < 0.001), including rates of intra-operative haemorrhage (RR 9.5; *p* < 0.001) and bowel injury (RR 40.6, *p* < 0.001). SLC was more likely to be performed in the emergency setting compared to TLC, had a longer operative time (125 vs. 73 min, *p* < 0.001) and both higher rates of intra-operative cholangiogram (RR 7.6; *p* < 0.001) and intra-operative drain insertion (RR 21.4, *p* < 0.001). There was no difference in time from admission to operation between the TLC and SLC groups.

**Table 4:** Adverse post-operative outcomes of SLC and TLC; univariate analysis

Adverse post-operative outcome	TLC (n = 2650)	SLC (n = 87)	Relative risk in SLC	*p* value
Post-operative complications	141 (5.3)	40 (45.9)	8.6	<0.001
Bile leak	13 (0.5)	25 (28.7)	58.9	<0.001
Collection	45 (1.7)	18 (20.7)	12.2	<0.001
Retained stone	34 (1.3)	8 (9.2)	7.2	<0.001
Pneumonia	17 (0.6)	3 (3.4)	5.4	<0.001
Bile duct injury	4	0 (0.0)	–	
Remnant cholecystitis	0 (0.0)	2 (2.2)	–	0.001
Other	48 (1.8)	2 (2.3)	1.3	0.74
Post-operative imaging	228 (8.6)	33 (37.9)	4.4	<0.001
USS	82 (3.1)	4 (4.6)	1.5	0.43
CT	104 (3.9)	25 (28.7)	7.3	<0.001
MRCP	96 (3.6)	20 (23.0)	6.3	<0.001
Post-operative intervention	62 (2.3)	25 (28.7)	12.3	<0.001
ERCP	36 (1.4)	20 (23.0)	16.9	<0.001
Laparoscopy	11 (0.4)	5 (5.7)	13.8	<0.001
Laparotomy	4 (0.2)	2 (2.3)	15.2	<0.001
Radiologically guided drainage	4 (0.2)	5 (5.7)	38.1	<0.001
Other	4 (0.2)	0 (0.0)	–	1
Post-operative LOS (median)	1	6	–	<0.001
Prolonged post-operative LOS (≥3 days)	197 (7.4)	73 (83.9)	11.3	<0.001
Readmission	175 (6.6)	26 (29.9)	4.5	<0.001
Post-operative mortality	4 (0.2)	0 (0.0)	–	1

Rates of post-operative complication (Clavien-Dindo ≥ 2) (45.9%), imaging (37.9%) intervention (28.7%) and readmission (29.9%) were considerable following SLC and were significantly higher than TLC (*p* < 0.001). The median post-operative length of stay (LOS) following SLC was 6 days. Rates of further imaging and intervention were higher (RR 4.4 and 12.3; respectively; *p* < 0.001) as well as rates of prolonged PLOS (11.3; *p* < 0.001) and readmission (RR 4.5; *p* < 0.001) (Table 4). In comparison to TLC, SLC had a higher rate of post-operative complications (RR 8.6; *p* < 0.001), including rates of bile leak (RR 58.9; *p* < 0.001), collection (RR 12.2; *p* < 0.001), retained stones (RR 7.2; *p* < 0.001) and pneumonia (RR 5.4; *p* < 0.001) (Table 3). There was also one case of a liver abscess and one case of liver infarction in the SLC group.

The operative and post-operative findings were consistent in the multivariate logistic regression and the propensity analysis (Table 5). In both analyses, after adjusting for pre-operative variables, SLC was positively associated with intra-operative drains, intra-operative complication, post-operative complication/imaging/intervention, prolonged post-operative LOS and readmission (*p* < 0.05).

**Table 5:** Relationship between SLC and Adverse outcomes: multivariate logistic regression and propensity analysis

Adverse outcome	Multi-variate logistic regression		Propensity analysis	
	Odds ratio	*p* value	Coefficient	*p* value
Intra-operative drain insertion	66.7	<0.001	0.77	<0.001
Intra-operative complications	5.64	<0.001	0.125	<0.001
Post-operative complications	9.44	<0.001	0.342	<0.001
Prolonged LOS	41.06	<0.001	0.700	<0.001
Post-operative imaging	4.81	<0.001	0.179	0.005
Post-operative intervention	10.72	<0.001	0.212	<0.001
Readmisison	4.65	<0.001	0.134	0.027

## Discussion

The present study demonstrates significant rates of peri-operative adverse outcomes following SLC and a heightened risk profile compared to that of TLC. Almost 50% of SLC patients suffer a post-operative complication, 28.7% require re-intervention, post-operative length of stay is significant (median, 6 days) and readmission is frequent (29.9%). Whilst SLC is justified to reduce the likelihood of ductal injury in difficult cholecystectomy, the disparate peri-operative course of SLC and TLC patients and the implications must be recognised.

The particular risk factors for SLC highlighted in this study are mostly expected. Higher age, male gender and previous cholecystitis are recognised risk factors for a difficult cholecystectomy; similarly, Farrugia et al. found pre-operative ERCP to be a risk factor for subtotal cholecystectomy [3–5, 10]. Less emphasised in the literature is the relationship between increased episodes of biliary-related attacks and difficult cholecystectomy, as demonstrated here. Recognising the number of episodes, particularly those severe enough to cause admission, has an important role in risk stratification as well as informed patient consent. Elective theatre list cancellations amidst the COVID pandemic, has led to increased waiting times and recurrent admissions in our unit. Perhaps the rate of subtotal cholecystectomy and other salvage techniques has increased consequently; however, further research is required.

The significant peri-operative morbidity associated with SLC can partly be explained by severe acute or chronic cholecystitis making dissection particularly challenging, risking intra-operative complications (e.g. haemorrhage, liver laceration, bile leak), further imaging and intervention. It can also be explained by the very nature of the procedure. The technique leaves Hartmann’s pouch in situ which is often left open. Both bile leaks and retained stones are a natural consequence, frequently requiring further imaging (MRCP) or intervention (ERCP) [11–14].

More specifically, there are a number of variations in SLC technique influencing peri-operative outcomes. Strasberg et al. described the ‘Fenestrated’ and ‘Reconstituting’ subtypes, whereby the gallbladder remnant is either left open or closed, respectively [15]. Purzner et al. expanded of this classification scheme by describing if the posterior wall has been left in situ [16]. A free omental plug over the gallbladder stump to close the stump has also been utilised successfully to reduce the rate of post-operative bile leak [17]. The Harilingam approach involves dissection of the anterior gallbladder wall to gain an internal view of the cystic duct to delineate the ductal anatomy, followed by an intracorporeal suture to close the remnant [18]. Overall, the closure of the remnant gallbladder is a major consideration. Henneman et al. found a reduced rate of bile leak following reconstituted subtotal versus a fenestrated subtotal (5.3% vs. 16.3%, respectively) and a reduced requirement for post-operative ERCP (2.7% vs. 16.0%) [19]. In our cohort, 83.9% had a fenestrated subtotal which may explain the significant rate of bile leak and post-operative ERCP. Lower morbidity has been suggested following reconstituting SLC; however, one must appreciate the risks of recurrent cholecystitis and the risk of ductal injury when closing the stump [9, 20].

Regardless of technique, several pertinent points apply. It is important to remove as many residual stones in the gallbladder stump which may require exploration of the cavity of gallbladder remnant if safe to do so [21]. Whilst removing stones, spillage should be minimised which can be a focus for post-operative collections. The gallbladder remnant should also be explored for the cystic duct and if identified then an intra-operative cholangiogram can be performed. Where CBD stones are identified, an attempt can be made to extract them intra-operatively or an early post-operative ERCP can be planned [19]. Whilst one may expect leaving posterior gallbladder wall in situ would precipitate further problems, post-operative morbidity is not higher in these cases and leaving the wall in situ avoids further complication (e.g. liver injury, significant haemorrhage) [9, 22]. In our cohort, 71.3% (62/87) had a posterior wall left in situ and we do not anticipate that this alone contributed to post-operative morbidity.

In cases where the critical view of safety cannot be established, the surgeon may consider other salvage techniques. Fundus-first dissection offers an alternative retrograde dissection technique which has been performed safely by many institutions with the possibility of removing the entire gallbladder. However, there is an established concern that fundus first dissection subjects the patient to a higher risk of ductal injury [23–27]. Less invasive techniques must also be considered. Endoscopic gallbladder stenting (EGS) has proved successful in elderly patients. Maekawa et al. reported low rates of recurrent cholecystitis (96.7%) and found the majority of patients to remain asymptomatic (93.5%) following the procedure [28]. Recurrent cholecystitis rates are lower following EGS compared percutaneous gallbladder drainage (0% vs. 17.2%, respectively), another less invasive technique [29]. Conservative management must also be considered. This has proved successful in acute cholecystitis, with 78% of patients remaining asymptomatic during long-term follow-up [30].

Recent literature reports reducing rates of conversion-to-open as SLC has gained popularity, particularly in face of difficult dissection of the cholecystohepatic triangle [8, 9, 31]. The present study corroborates this finding, where the conversion rate was 1.1%, suggesting that SLC is increasingly becoming the default salvage option. Interestingly, institutions have observed a decline in the rates of iatrogenic ductal injury as the concept of SLC has gained traction [8, 31]. Certainly, the present study confirms that SLC can be performed in challenging cases without a significant risk of BDI. Whilst the overall morbidity of laparoscopic subtotal is lower than open subtotal, it remains unclear the proportion of difficult cholecystectomies which would proceed to a total cholecystectomy if converted to open [9]. The paradigm shift in favour of SLC may be driven by familiarity with laparoscopic salvage options and inexperience with open cholecystectomy, instead of favourable outcomes [22, 32].

The present data indicate the importance of discussing the implications of a SLC with high-risk patients as part of an informed consent process. The risk profile of a SLC is entirely different to that of TLC. Particular notable findings include the significant risk of post-operative complication (45.9%), readmission (29.9%), re-intervention (28.7%) and longer length of stay (median, 6 days). In fact, bile leak was 58.9 times more likely, collections 12.2 times more likely and retained stones 7.2 times more likely following SLC. Clearly the standard consent discussion is not adequate to prepare the patient for the likely peri-operative course if a subtotal cholecystectomy is performed. Of course, discussing the implications of SLC is far more relevant in high-risk patients, such as those with multiple episodes of cholecystitis, previous ERCP or even active inflammation, but less pertinent to elective cholecystectomy patients with simple biliary colic.

Although this study reports post-operative outcomes for 100 days, this does not consider overall quality of life (QOL) related to the procedure. QOL studies are required to compare overall quality of life of an SLC with alternatives such as conversion-to-open. For example, SLC patients may have to tolerate intra-operative drains draining bile for weeks or may suffer additional complications secondary to ERCP. Chronic pain following SLC patients may also be a serious concern but has not been investigated.

In conclusion, the relative morbidity associated with SLC compared to TLC is significant and has implications for consent in high-risk patients and intra-operative decision making. Whilst SLC is justified to reduce the likelihood of ductal injury in difficult cholecystectomy, the disparate peri-operative course of SLC compared to TLC and its implications must be recognised. Prospective studies comparing the outcomes of salvage techniques and less invasive techniques (e.g. EGS, cholecystostomy) in particular subgroups will help clarify the role of subtotal in face of the difficult cholecystectomy.
